# Effect of reminder letters after health checkups on the consultation behavior of participants with possible hypertension, hyperglycemia, and dyslipidemia: A retrospective cohort study using administrative claims data in Japan

**DOI:** 10.1002/1348-9585.12231

**Published:** 2021-05-11

**Authors:** Nobuaki Hoshino, Mo Xiuting, Yoshimitsu Takahashi, Takeo Nakayama

**Affiliations:** ^1^ Department of Health Informatics School of Public Health Graduate School of Medicine Kyoto University Kyoto Japan; ^2^ Department of Surgery Kyoto University Graduate School of Medicine Kyoto Japan

**Keywords:** consultation behavior, health checkups, lifestyle disease, noncommunicable disease, reminder

## Abstract

**Objectives:**

Prevention of and early treatment for noncommunicable diseases such as hypertension, hyperglycemia, and dyslipidemia are important, as these diseases are asymptomatic in early stages but can lead to critical conditions such as macro‐ and microvascular disorders later on. While screening is conducted worldwide, low rates of hospital visits after screening is a common issue. We aimed to investigate the effect of reminder letters on the consultation behavior of screened participants.

**Methods:**

We used administrative claims data from a database managed by JMDC Inc for participants of health checkups in 2014, 2015, 2016, and 2017, who belonged to a health insurance society. Reminder letters were sent regularly 6 months after checkups to improve participant consultation behavior. Participants who screened positive for hypertension, hyperglycemia, and dyslipidemia, and who were not taking medication for any of these diseases at the time of health checkups, were included in the analyses.

**Results:**

A total of 1739 participants in 2014, 1693 in 2015, 2002 in 2016, and 2144 in 2017 were included in the analysis for hypertension. The cumulative proportion of hospital visits gradually increased over the course of 12 months after checkups in all years. After 2015, spikes, albeit very small ones, were observed at 6 months after checkups in accordance with the timing of reminder letters. Similar trends were observed for hyperglycemia and dyslipidemia.

**Conclusions:**

Sending reminder letters is a potentially effective approach to increase hospital visits, but further improvements (ie, multiple reminders) may be necessary to affect enhancements in participant consultation behavior.

## 1 INTRODUCTION

Noncommunicable diseases (NCDs) are asymptomatic in early stages but can lead to cardiovascular, cerebral, and renal diseases if advanced.^1^ NCDs are often referred to as lifestyle diseases, which are defined as diseases that develop due to unhealthy daily habits and diet. Lifestyle diseases are preventable, and modifiable factors for prevention include hypertension, hyperglycemia, and dyslipidemia.^1^ Specifically, the prevention of and adequate treatment for hypertension, hyperglycemia, and dyslipidemia can lead to decreased rates of critical diseases.

Screening for NCDs is conducted in many countries, including both developed and developing countries.^2^, ^3^ In Japan, annual health checkups are promoted under the universal health insurance system for the prevention and early detection of hypertension, hyperglycemia, and dyslipidemia.^4^ In April 2008, the Ministry of Health, Labour and Welfare initiated specific health checkups focused on visceral adiposity in order to reduce lifestyle diseases. However, while a checkup examination is offered every year, only 20%‐30% of participants with possible hypertension, hyperglycemia, or dyslipidemia subsequently visit a hospital after screening.^5^, ^6^ In addition to individual approaches to modify lifestyles, group or systematic approaches are desired for the better management of lifestyle diseases.

In order to increase consultation rates after health checkups, health insurance societies began sending reminder letters to screened participants who failed to follow‐up for possible hypertension, hyperglycemia, or dyslipidemia in 2014. However, it is unclear whether these reminder letters had a positive effect on the consultation behavior of screened participants. In this study, we aimed to investigate the effect of these reminder letters on the consultation behavior of screened participants with possible hypertension, hyperglycemia, or dyslipidemia.

## 2 MATERIALS AND METHODS

### 2.1 Study design and setting

This study was a retrospective cohort study using administrative claims data from JMDC Inc. The company collects data on both health checkups and health insurance claims from several employee‐based social health insurance plans and stores them in the JMDC medical database.^7^ As of April 2020, the database contained data for 7.3 million cumulative insured individuals, mainly company employees and their family members.^8^ In Japan, health checkups are conducted on a fiscal‐year basis (fiscal year [FY] in Japan starts in April and ends in March). Health insurance societies that began sending reminder letters in FY 2015 were targeted in this study. Data collected before (2014) and after (2015 and thereafter) the reminder letter practice began were compared. The reminder letters were sent by mail from occupational health staffs of each health insurance society. Participants will take no disadvantages unless they visit hospital after receiving the mail. This study was approved by the Ethics Committee of Kyoto University (E1017).

### 2.2 Timing of reminder letters

We examined data from eight health insurance societies that began sending reminder letters in FY 2015. Of these, one society has been sending reminder letters regularly, around 6 months after health checkups, every year since FY2015. The remaining seven societies have been sending reminder letters at irregular intervals of 6‐19 months after health checkups since FY 2015. We excluded the health insurance societies with irregular reminders and restricted our analysis to the health insurance society with regular reminders, as the present study focused on the effect of regular reminder letters given that health checkups are conducted on a regular basis every FY. During the study period, 22 162, 21 074, 24 526, and 25 580 participants underwent health checkups in FY 2014, 2015, 2016, and 2017, respectively.

### 2.3 Definitions of hypertension, hyperglycemia, and dyslipidemia in health checkups

Blood pressure was measured after the participant rested for several minutes in a sitting position. In the Japanese Society of Hypertension Guideline for the Management of Hypertension 2019, hypertension is categorized as follows: Class I, systolic blood pressure (SBP) 140‐159 mm Hg and/or diastolic blood pressure (DBP) 90‐99 mm Hg; Class II, SBP 160‐179 mm Hg and/or DBP 100‐109 mm Hg; and Class III, SBP ≥180 mm Hg and/or ≥DBP 110 mm Hg.^9^ In this study, participants were screened for Class II hypertension to identify those who potentially require medication or health guidance. Glycated hemoglobin (HbA_1c_) ≥6.5% and/or fasting glucose ≥126 mg/dL were used to screen for possible hyperglycemia according to the Japanese Clinical Practice Guideline for Diabetes 2016.^10^ Triglyceride ≥300 mg/dL (regardless of fasting status), low density lipoprotein‐cholesterol ≥140 mg/dL, and/or high density lipoprotein‐cholesterol <35 mg/dL were used to screen for possible dyslipidemia.

### 2.4 Study participants

The participants of this study were composed of workers and their families. Most of the workers engaged in manufacturing industries. Participants who underwent health checkups from FY 2014 to 2017 were considered eligible for this study. Participants who screened positive for hypertension, hyperglycemia, and/or dyslipidemia and were taking no medication for any of these diseases were included in the analyses.

### 2.5 Outcome measures

The number of first hospital visits during the 12 months after health checkups was counted. A hospital visit was defined as the presence of any ICD‐10 code suggesting hypertension (I10‐I15), hyperglycemia (E10‐E14), or dyslipidemia (E78) in each month.

### 2.6 Statistical analysis

The number and proportion in each month of participants who visited a hospital within 12 months after health checkups were determined. Cumulative proportions of participants were plotted, and descriptive analysis was performed. The cumulative proportion of participants in FY 2014 was set as a reference for comparisons with cumulative proportions in FY 2015‐2017.

## 3 RESULTS

### 3.1 Hypertension

**FIGURE 1:**
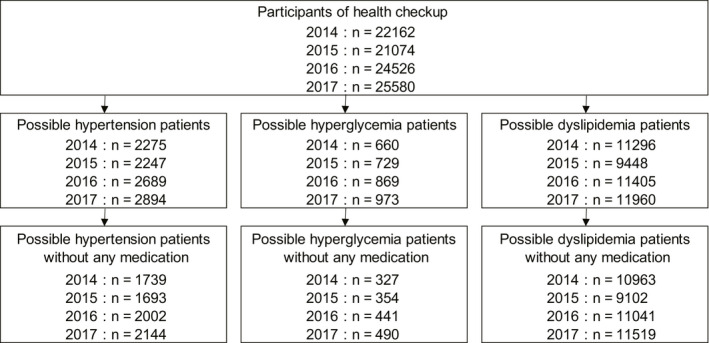
Flow diagram of participant selection: hypertension, hyperglycemia, and dyslipidemia

During the study period, 1739, 1693, 2002, and 2144 participants in FY 2014, 2015, 2016, and 2017, respectively, were included in the analysis (Figure 1).

**FIGURE 2:**
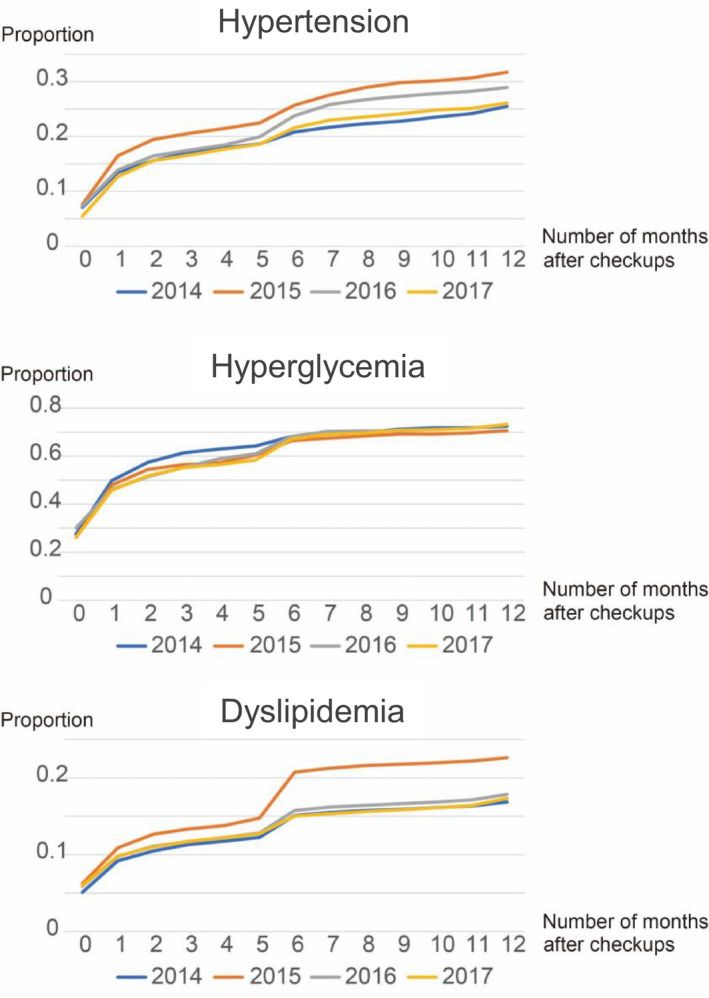
Cumulative proportion of hospital visits within 12 months after health checkups. Total numbers of study participants in FY 2014, 2015, 2016, and 2017 were 1739, 1693, 2002, and 2144, respectively, in hypertension, 327, 354, 441, and 490, respectively, in hyperglycemia, and 10 963, 9102, 11 041, and 11 519, respectively, in dyslipidemia

Numbers of participants who visited a hospital within 12 months after health checkups are shown in Table S1, and their cumulative proportions are shown in Figure 2A. Although the curve gradually increased in FY 2014, a small spike in the number of hospital visits was noted at 6 months after health checkups in FY 2015, 2016, and 2017. Cumulative proportions at 12 months after health checkups in FY 2015, 2016, and 2017 were higher than that in FY 2014.

### 3.2 Hyperglycemia

During the study period, 327, 354, 441, and 490 participants in FY 2014, 2015, 2016, and 2017, respectively, were included in the analysis (Figure 1).

Numbers of participants who visited a hospital 12 months after health checkups are shown in Table S2, and their cumulative proportions are shown in Figure 2B. Although a small spike in the number of hospital visits was noted at 6 months after health checkups in FY 2015, 2016, and 2017, cumulative proportions were similar across all years.

### 3.3 Dyslipidemia

During the study period, 10 963, 9102, 11 041, and 11 519 participants in FY 2014, 2015, 2016, and 2017, respectively, were included in the analysis (Figure 1).

Numbers of participants who visited a hospital within 12 months after health checkups are shown in Table S3, and their cumulative proportions are shown in Figure 2C. Although a spike at 6 months after health checkups was noted in FY 2015, trends for FY 2016 and 2017 were similar to those of FY 2014. The cumulative proportion at 12 months after health checkups was higher in FY 2015 compared to FY 2014, whereas no differences were observed in cumulative proportions among FY 2014, 2016, and 2017.

## 4 DISCUSSION

In this study, we investigated the effect of regular reminder letters on the consultation behavior of screened participants with possible hypertension, hyperglycemia, and dyslipidemia who did not visit a hospital after health checkups. The number of hospital visits at 6 months after health checkups slightly increased after FY 2015 for all diseases. Only a small increase in cumulative proportion was observed at 12 months after health checkups for hypertension and dyslipidemia. It was unclear whether reminder letters after health checkups improved consultation rates among patients potentially dealing with lifestyle diseases.

The US Preventive Service Task Force recommends screening for high blood pressure in all adults, and screening for diabetes in middle‐aged and older individuals with any risk factors, such as obesity and a family history of diabetes.^11^, ^12^ Meanwhile, there are currently no reports regarding the effect of screening for dyslipidemia in younger adults.^13^ In Japan, the government began offering Specific Health Checkups and Specific Health Guidance in 2008, in which middle‐aged adults (aged 40‐74 years) undergo screening for hypertension, hyperglycemia, and dyslipidemia for the prevention and early detection of lifestyle diseases. However, approximately 80% of participants with possible hypertension, and 65% of participants with possible hyperglycemia, fail to follow‐up within 6 months after health checkups.^5^, ^6^ In Japan, participants are free to visit any hospital they prefer under the free access system.^4^, ^14^ Doctors at health checkups do not follow up on participants, even if they are found to be at high risk of developing lifestyle diseases during health checkups.^5^ In other words, it is up to participants themselves to consult a doctor after their checkups. Although screening is conducted in many countries including Japan, the effects of screening for hypertension, hyperglycemia, or dyslipidemia have not been fully examined.^2^, ^3^, ^13^ Low consultation rates after health checkups might contribute to the lack of efficacy of screening in Japan.

In this study, the proportion of participants who visited a hospital within 12 months after checkups for possible hypertension was 25.8%‐31.2%, which is comparable to a previously reported rate.^5^ As for participants with possible hyperglycemia, the proportion was 70.6%‐73.3%, also comparable to a previously reported rate.^6^ In other words, consultation rates for both possible hypertension and possible hyperglycemia have not improved relative to previous reports. The goal of treating hypertension, hyperglycemia, and dyslipidemia goes beyond controlling blood pressure, blood glucose, blood triglycerides, or blood cholesterol; it is to prevent the development of critical diseases. Moreover, the control of blood pressure, blood glucose, blood triglyceride, and blood cholesterol can mutually influence the progression of each of these.^9^, ^10^ Ultimately, what is important is increasing the number of screened participants who follow‐up for possible lifestyle diseases.

Even if health checkups for hypertension, hyperglycemia, and dyslipidemia are effectively carried out, these efforts are wasted if participants with possible diseases fail to follow‐up for consultation. It remains unclear how to motivate participants with possible lifestyle diseases to visit a hospital. The present study examined the effect of regular reminder letters at 6 months after health checkups, which are conducted every FY. Some health insurance societies send reminder letters more than 12 months after health checkups. This is unlikely to be effective, since the 12‐month interval is too long and the next health checkup might have already been conducted. Although the present study did not observe the effect of reminder letters on participant consultation behavior, a small increase in the number of hospital visits was noted around the time the letters were sent. That is to say, there was no spike at 6 months after health checkups for all diseases in FY 2014 while there were small spikes after FY 2015 in Figure 2, although the proportion of hospital visits after 6 months after health checkups was almost same for hyperglycemia in all years. In recent years, an increasing number of insurance societies began using improved methods such as illustrated reminder cards. Accordingly, investigating the effectiveness of reminder letters from multiple insurance societies might be informative.

The strength of this study is that health checkup data were linked to medical data. In Japan, doctors at health checkups usually differ from those who provide medical treatment. Thus, it is rare to include both health checkup data and treatment data in one study. However, the present study also has some limitations. First, a randomized trial is more ideal to determine the effect of reminder letters on participant consultation behavior, since time series comparisons performed in the present study are subject to bias, such as the period effect. Second, participants of the present study were covered by employee‐based social insurance and included more middle‐aged people than older people. Finally, this study used data from a single health insurance society, and thus, generalizability of the present findings may be limited. Moreover, this health intervention should be evaluated from viewpoints of cost‐effectiveness ideally.

## 5 CONCLUSION

Only a small increase in the number of hospital visits was noted around the time reminder letters were sent. Sending reminder letters is a potentially effective approach to increase hospital visits, but further upgrades such as multiple reminders may be needed to affect improvements in participant consultation behavior.

## DISCLOSURE

*Approval of the research protocol*: This study was approved by the Ethics Committee of Kyoto University (E1017). *Informed consent*: N/A. *Registry and the registration no. of the study*: N/A. *Animal studies*: N/A. *Conflict of interest*: Takeo Nakayama has received personal fees from Otsuka Pharmaceutical Co., Nakamura Hospital, JMDC Inc, Dainippon Sumitomo Pharmaceutical Co., Ono Pharmaceutical Co., Chugai Pharmaceutical Co., Dentsu Co., Takeda Pharmaceutical Co., Novo Nordisk Pharma Co., Janssen Pharmaceutical KK, Boehringer Ingelheim International GmbH, HANSHIN Dispensing Holding Co. Ltd., Pfizer Japan Inc, Nikkei Business Publications, Inc, Eli Lilly Japan KK, Baxter, and Alexion outside the submitted work. The remaining authors have no conflicts of interest to disclose.

## AUTHOR CONTRIBUTIONS

All authors conceived the idea of this study; YH and TN collected the data; NH and MX analyzed the data; and all authors contributed to the writing of the manuscript.

## Supporting information

Supplementary Material
