# Supplementary material for: Effect of reminder letters after health checkups on the consultation behavior of participants with possible hypertension, hyperglycemia, and dyslipidemia: A retrospective cohort study using administrative claims data in Japan
Source: J Occup Health. 2021 May 11;63(1):e12231. doi: 10.1002/1348-9585.12231 (PMC8112116; doi:10.1002/1348-9585.12231)
Supplement: Supplementary file 1 — Supplementary Material [file JOH2-63-e12231-s001.docx]

**Appendices**

| Table S1 Number of hospital visits after health checkups: hypertension | | | | | | | | |
| --- | --- | --- | --- | --- | --- | --- | --- | --- |
| Time after health checkups (months) | Hospital visits | | | | | | | |
|  | 2014 | | 2015 | | 2016 |  | 2017 | |
|  | n | % | n | % | n | % | n | % |
| 0 | 123 | 27.8 | 129 | 24.0 | 146 | 25.2 | 117 | 20.9 |
| 1 | 111 | 25.1 | 150 | 27.9 | 132 | 22.8 | 155 | 27.7 |
| 2 | 37 | 8.4 | 50 | 9.3 | 51 | 8.8 | 62 | 11.1 |
| 3 | 27 | 6.1 | 19 | 3.5 | 21 | 3.6 | 21 | 3.8 |
| 4 | 14 | 3.2 | 15 | 2.8 | 19 | 3.3 | 24 | 4.3 |
| 5 | 12 | 2.7 | 17 | 3.2 | 30 | 5.2 | 19 | 3.4 |
| 6 | 38 | 8.6 | 55 | 10.2 | 77 | 13.3 | 65 | 11.6 |
| 7 | 15 | 3.4 | 32 | 6.0 | 41 | 7.1 | 30 | 5.4 |
| 8 | 11 | 2.5 | 23 | 4.3 | 18 | 3.1 | 12 | 2.1 |
| 9 | 8 | 1.8 | 15 | 2.8 | 12 | 2.1 | 12 | 2.1 |
| 10 | 13 | 2.9 | 5 | 0.9 | 10 | 1.7 | 15 | 2.7 |
| 11 | 11 | 2.5 | 9 | 1.7 | 8 | 1.4 | 6 | 1.1 |
| 12 | 23 | 5.2 | 18 | 3.4 | 14 | 2.4 | 22 | 3.9 |

| Table S2 Number of hospital visits after health checkups: hyperglycemia | | | | | | | | |
| --- | --- | --- | --- | --- | --- | --- | --- | --- |
| Time after health checkups (months) | Hospital visits | | | | | | | |
|  | 2014 | | 2015 | | 2016 | | 2017 | |
|  | n | % | n | % | n | % | n | % |
| 0 | 90 | 38.0 | 93 | 37.2 | 133 | 41.2 | 128 | 35.7 |
| 1 | 73 | 30.8 | 77 | 30.8 | 71 | 22.0 | 97 | 27.0 |
| 2 | 25 | 10.5 | 23 | 9.2 | 23 | 7.1 | 29 | 8.1 |
| 3 | 13 | 5.5 | 7 | 2.8 | 17 | 5.3 | 17 | 4.7 |
| 4 | 5 | 2.1 | 3 | 1.2 | 16 | 5.0 | 6 | 1.7 |
| 5 | 4 | 1.7 | 11 | 4.4 | 9 | 2.8 | 9 | 2.5 |
| 6 | 13 | 5.5 | 21 | 8.4 | 32 | 9.9 | 43 | 12.0 |
| 7 | 3 | 1.3 | 4 | 1.6 | 9 | 2.8 | 10 | 2.8 |
| 8 | 2 | 0.8 | 3 | 1.2 | 1 | 0.3 | 2 | 0.6 |
| 9 | 5 | 2.1 | 3 | 1.2 | 0 | 0.0 | 6 | 1.7 |
| 10 | 2 | 0.8 | 0 | 0.0 | 3 | 0.9 | 1 | 0.3 |
| 11 | 0 | 0.0 | 2 | 0.8 | 2 | 0.6 | 3 | 0.8 |
| 12 | 2 | 0.8 | 3 | 1.2 | 7 | 2.2 | 8 | 2.2 |

| Table S3 Number of hospital visits after health checkups: dyslipidemia | | | | | | | | |
| --- | --- | --- | --- | --- | --- | --- | --- | --- |
| Time after health checkups (months) | Hospital visits | | | | | | | |
|  | 2014 | | 2015 | | 2016 | | 2017 | |
|  | n | % | n | % | n | % | n | % |
| 0 | 559 | 30.3 | 567 | 27.6 | 650 | 33.0 | 688 | 34.5 |
| 1 | 450 | 24.4 | 423 | 20.6 | 427 | 21.7 | 443 | 22.2 |
| 2 | 139 | 7.5 | 161 | 7.8 | 151 | 7.7 | 137 | 6.9 |
| 3 | 92 | 5.0 | 63 | 3.1 | 62 | 3.1 | 84 | 4.2 |
| 4 | 48 | 2.6 | 40 | 1.9 | 61 | 3.1 | 56 | 2.8 |
| 5 | 54 | 2.9 | 88 | 4.3 | 64 | 3.2 | 58 | 2.9 |
| 6 | 313 | 17.0 | 546 | 26.5 | 322 | 16.3 | 265 | 13.3 |
| 7 | 43 | 2.3 | 46 | 2.2 | 51 | 2.6 | 35 | 1.8 |
| 8 | 27 | 1.5 | 31 | 1.5 | 26 | 1.3 | 31 | 1.6 |
| 9 | 19 | 1.0 | 16 | 0.8 | 22 | 1.1 | 28 | 1.4 |
| 10 | 25 | 1.4 | 18 | 0.9 | 25 | 1.3 | 29 | 1.5 |
| 11 | 21 | 1.1 | 19 | 0.9 | 28 | 1.4 | 35 | 1.8 |
| 12 | 56 | 3.0 | 39 | 1.9 | 81 | 4.1 | 106 | 5.3 |
